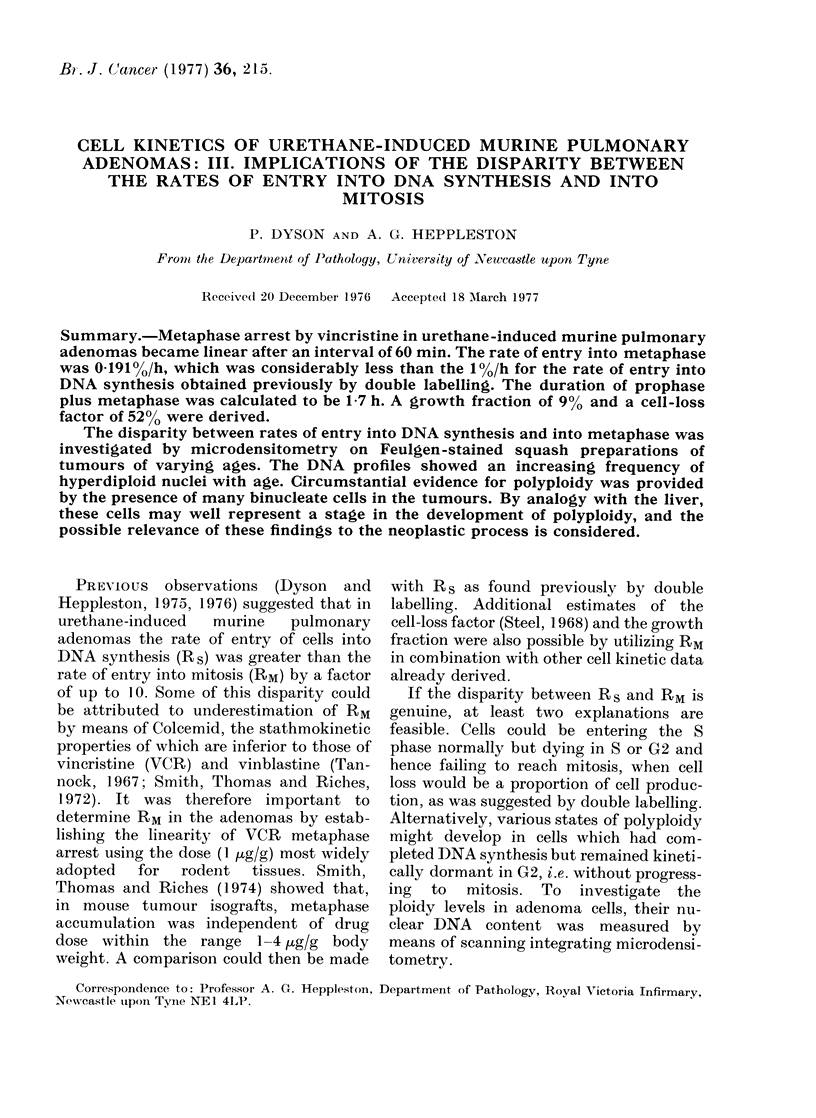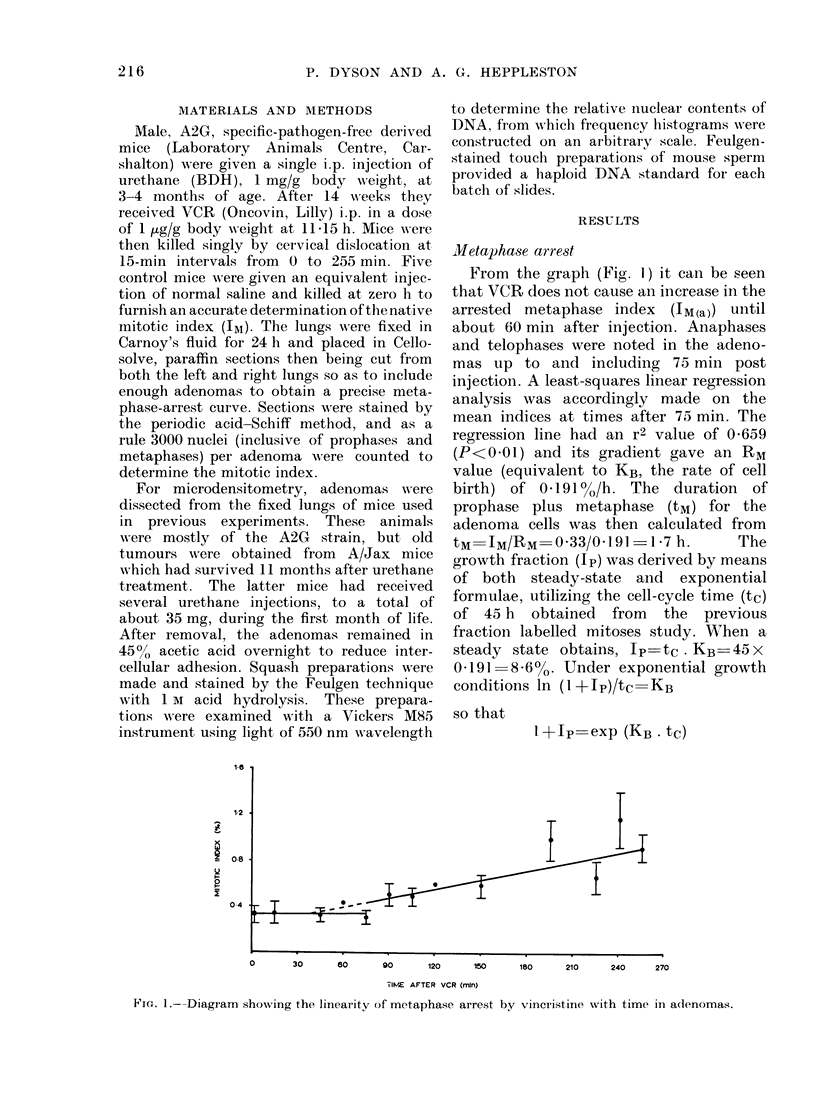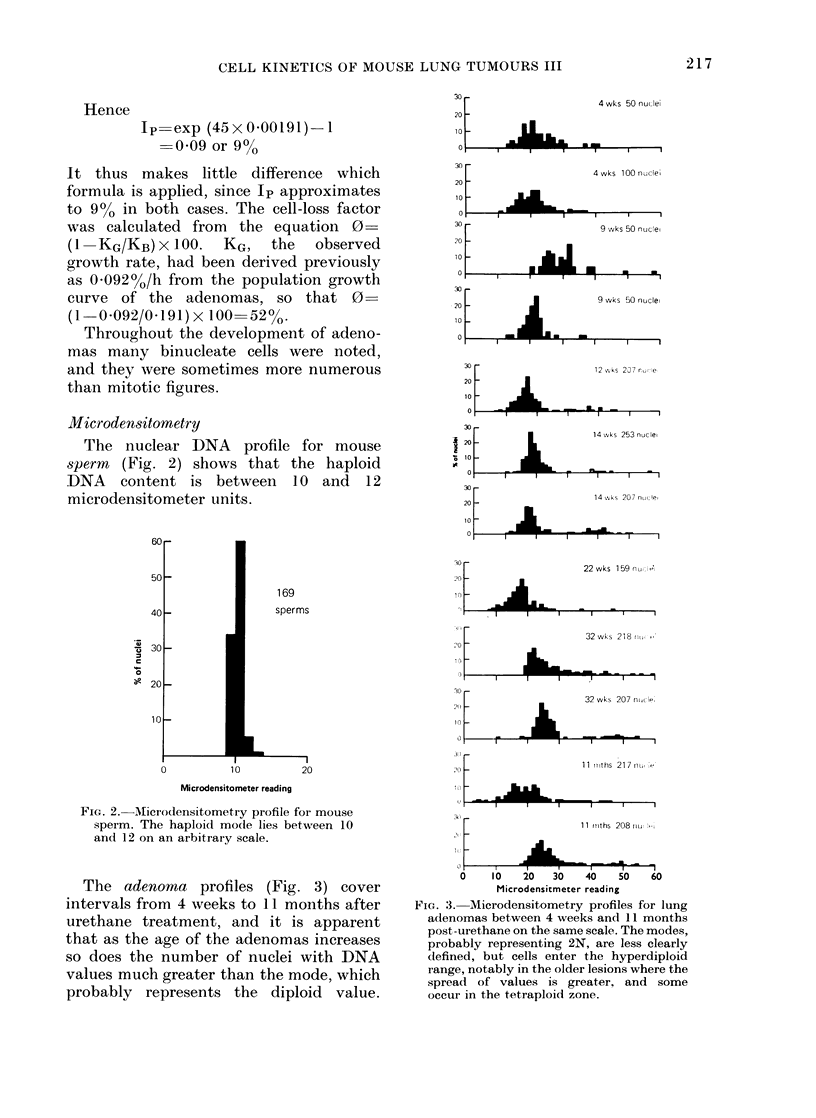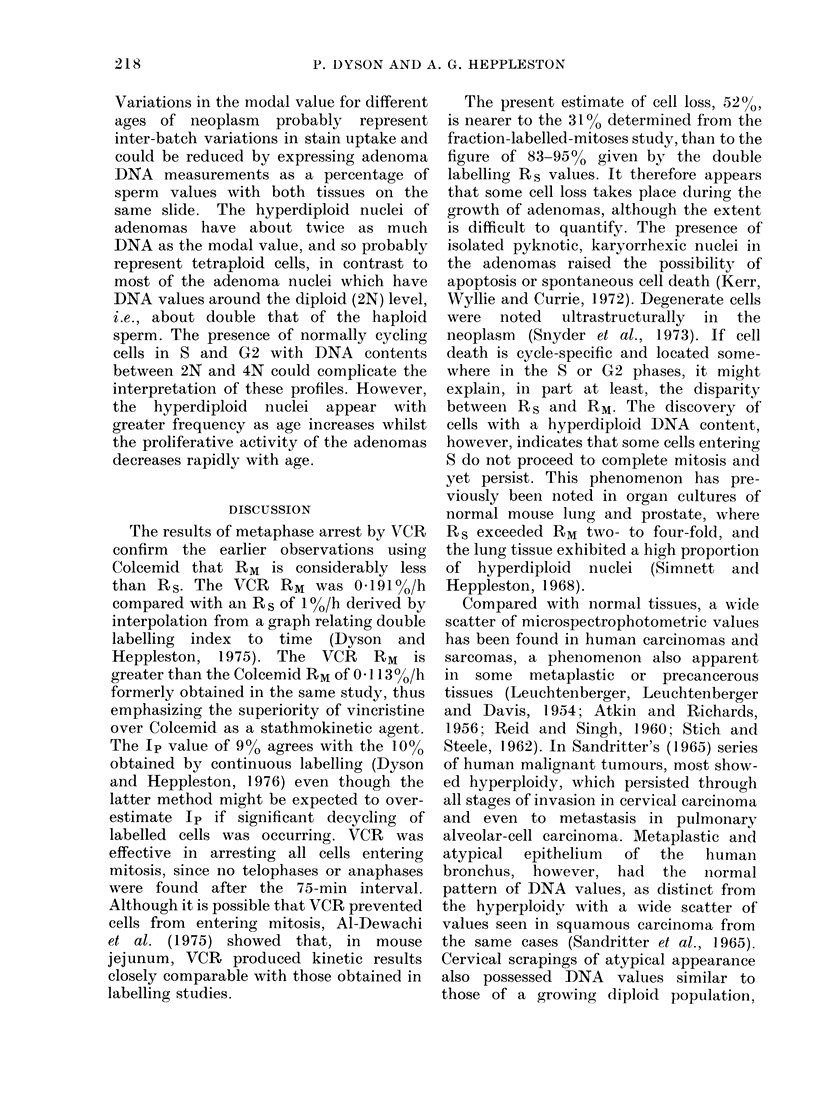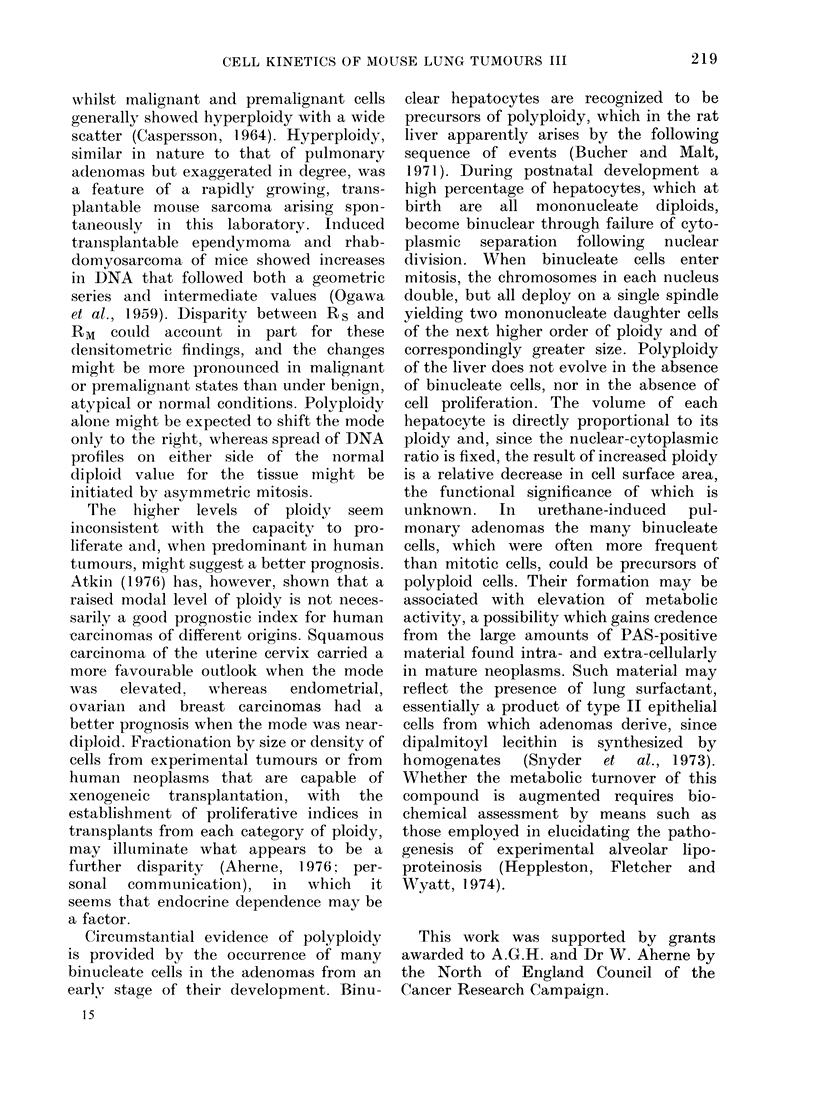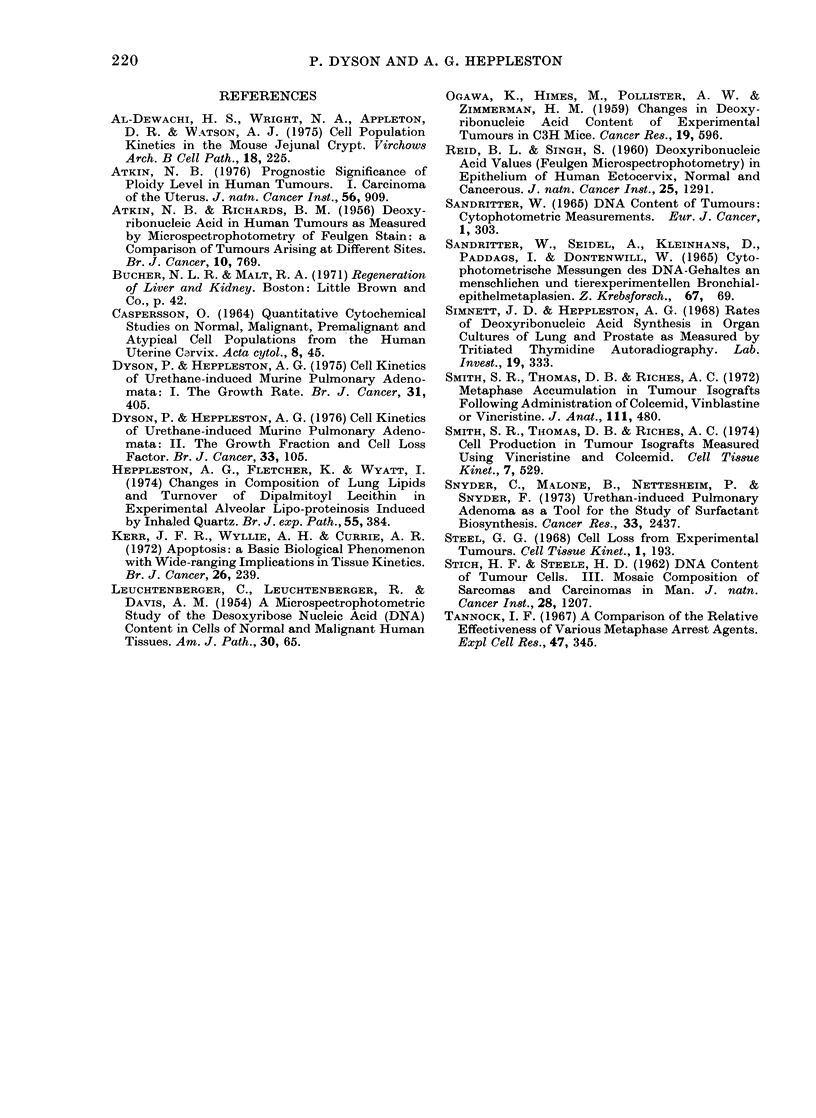# Cell kinetics of urethane-induced murine pulmonary adenomas: III. Implications of the disparity between the rates of entry into DNA synthesis and into mitosis.

**DOI:** 10.1038/bjc.1977.180

**Published:** 1977-08

**Authors:** P. Dyson, A. G. Heppleston

## Abstract

Metaphase arrest by vincristine in urethane-induced murine pulmonary adenomas became linear after an interval of 60 min. The rate of entry into metaphase was 0-191%/h, which was considerably less than the 1%/h for the rate of entry into DNA synthesis obtained previously by double labelling. The duration of prophase plus metaphase was calculated to be 1-7 h. A growth fraction of 9% and a cell-loss factor of 52% were derived. The disparity between rates of entry into DNA synthesis and into metaphase was investigated by microdensitometry on Feulgen-stained squash preparations of tumours of varying ages. Tne DNA profiles showed an increasing frequency of hyperdiploid nuclei with age. Circumstantial evidence for polyploidy was provided by the presence of many binucleate cells in the tumours. By analogy with the liver, these cells may well represent a stage in the development of polyploidy, and the possible relevance of these findings to the neoplastic process is considered.


*Br. J. Cancer* (1977) **36**, 215.

### CELL KINETICS OF URETHANE-INDUCED MURINE PULMONARY ADENOMAS: III. IMPLICATIONS OF THE DISPARITY BETWEEN THE RATES OF ENTRY INTO DNA SYNTHESIS AND INTO MITOSIS

P. DYSON AND A. G. HEPPLESTON

*From the Department of Pathology, University of Newcastle upon Tyne*

Received 20 December 1976 Accepted 18 March 1977

**Summary.—Metaphase arrest by vincristine in urethane-induced murine pulmonary adenomas became linear after an interval of 60 min. The rate of entry into metaphase was 0·191%/h, which was considerably less than the 1%/h for the rate of entry into DNA synthesis obtained previously by double labelling. The duration of prophase plus metaphase was calculated to be 1·7 h. A growth fraction of 9% and a cell-loss factor of 52% were derived.**

**The disparity between rates of entry into DNA synthesis and into metaphase was investigated by microdensitometry on Feulgen-stained squash preparations of tumours of varying ages. The DNA profiles showed an increasing frequency of hyperdiploid nuclei with age. Circumstantial evidence for polyploidy was provided by the presence of many binucleate cells in the tumours. By analogy with the liver, these cells may well represent a stage in the development of polyploidy, and the possible relevance of these findings to the neoplastic process is considered.**

PREVIOUS observations (Dyson and Heppleston, 1975, 1976) suggested that in urethane-induced murine pulmonary adenomas the rate of entry of cells into DNA synthesis ($R_S$) was greater than the rate of entry into mitosis ($R_M$) by a factor of up to 10. Some of this disparity could be attributed to underestimation of $R_M$ by means of Colcemid, the stathmokinetic properties of which are inferior to those of vincristine (VCR) and vinblastine (Tannock, 1967; Smith, Thomas and Riches, 1972). It was therefore important to determine $R_M$ in the adenomas by establishing the linearity of VCR metaphase arrest using the dose (1 μg/g) most widely adopted for rodent tissues. Smith, Thomas and Riches (1974) showed that, in mouse tumour isografts, metaphase accumulation was independent of drug dose within the range 1–4 μg/g body weight. A comparison could then be made with $R_S$ as found previously by double labelling. Additional estimates of the cell-loss factor (Steel, 1968) and the growth fraction were also possible by utilizing $R_M$ in combination with other cell kinetic data already derived.

If the disparity between $R_S$ and $R_M$ is genuine, at least two explanations are feasible. Cells could be entering the S phase normally but dying in S or G2 and hence failing to reach mitosis, when cell loss would be a proportion of cell production, as was suggested by double labelling. Alternatively, various states of polyploidy might develop in cells which had completed DNA synthesis but remained kinetically dormant in G2, *i.e.* without progressing to mitosis. To investigate the ploidy levels in adenoma cells, their nuclear DNA content was measured by means of scanning integrating microdensitometry.

Correspondence to: Professor A. G. Heppleston, Department of Pathology, Royal Victoria Infirmary, Newcastle upon Tyne NE1 4LP.

### MATERIALS AND METHODS

Male, A2G, specific-pathogen-free derived mice (Laboratory Animals Centre, Carshalton) were given a single i.p. injection of urethane (BDH), 1 mg/g body weight, at 3–4 months of age. After 14 weeks they received VCR (Oncovin, Lilly) i.p. in a dose of 1 $\mu$g/g body weight at 11·15 h. Mice were then killed singly by cervical dislocation at 15-min intervals from 0 to 255 min. Five control mice were given an equivalent injection of normal saline and killed at zero h to furnish an accurate determination of the native mitotic index ($I_M$). The lungs were fixed in Carnoy's fluid for 24 h and placed in Cellosolve, paraffin sections then being cut from both the left and right lungs so as to include enough adenomas to obtain a precise metaphase-arrest curve. Sections were stained by the periodic acid–Schiff method, and as a rule 3000 nuclei (inclusive of prophases and metaphases) per adenoma were counted to determine the mitotic index.

For microdensitometry, adenomas were dissected from the fixed lungs of mice used in previous experiments. These animals were mostly of the A2G strain, but old tumours were obtained from A/Jax mice which had survived 11 months after urethane treatment. The latter mice had received several urethane injections, to a total of about 35 mg, during the first month of life. After removal, the adenomas remained in 45% acetic acid overnight to reduce intercellular adhesion. Squash preparations were made and stained by the Feulgen technique with 1 M acid hydrolysis. These preparations were examined with a Vickers M85 instrument using light of 550 nm wavelength to determine the relative nuclear contents of DNA, from which frequency histograms were constructed on an arbitrary scale. Feulgen-stained touch preparations of mouse sperm provided a haploid DNA standard for each batch of slides.

### RESULTS

### *Metaphase arrest*

From the graph (Fig. 1) it can be seen that VCR does not cause an increase in the arrested metaphase index ($I_{M(a)}$) until about 60 min after injection. Anaphases and telophases were noted in the adenomas up to and including 75 min post injection. A least-squares linear regression analysis was accordingly made on the mean indices at times after 75 min. The regression line had an $r^2$ value of 0·659 ($P<0·01$) and its gradient gave an $R_M$ value (equivalent to $K_B$, the rate of cell birth) of 0·191%/h. The duration of prophase plus metaphase ($t_M$) for the adenoma cells was then calculated from $t_M=I_M/R_M=0·33/0·191=1·7$ h. The growth fraction ($I_P$) was derived by means of both steady-state and exponential formulae, utilizing the cell-cycle time ($t_C$) of 45 h obtained from the previous fraction labelled mitoses study. When a steady state obtains, $I_P=t_C \,.\, K_B=45\times 0·191=8·6\%$. Under exponential growth conditions $\ln\,(1+I_P)/t_C=K_B$

so that

$$1+I_P=\exp\,(K_B\,.\,t_C)$$

FIG. 1.—Diagram showing the linearity of metaphase arrest by vincristine with time in adenomas.

Hence

$$I_P = \exp(45 \times 0{\cdot}00191) - 1$$

$$= 0{\cdot}09 \text{ or } 9\%$$

It thus makes little difference which formula is applied, since $I_P$ approximates to 9% in both cases. The cell-loss factor was calculated from the equation $\emptyset = (1 - K_G/K_B) \times 100$. $K_G$, the observed growth rate, had been derived previously as 0·092%/h from the population growth curve of the adenomas, so that $\emptyset = (1 - 0{\cdot}092/0{\cdot}191) \times 100 = 52\%$.

Throughout the development of adenomas many binucleate cells were noted, and they were sometimes more numerous than mitotic figures.

### *Microdensitometry*

The nuclear DNA profile for mouse *sperm* (Fig. 2) shows that the haploid DNA content is between 10 and 12 microdensitometer units.

FIG. 2.—Microdensitometry profile for mouse sperm. The haploid mode lies between 10 and 12 on an arbitrary scale.

The *adenoma* profiles (Fig. 3) cover intervals from 4 weeks to 11 months after urethane treatment, and it is apparent that as the age of the adenomas increases so does the number of nuclei with DNA values much greater than the mode, which probably represents the diploid value.

FIG. 3.—Microdensitometry profiles for lung adenomas between 4 weeks and 11 months post-urethane on the same scale. The modes, probably representing 2N, are less clearly defined, but cells enter the hyperdiploid range, notably in the older lesions where the spread of values is greater, and some occur in the tetraploid zone.

Variations in the modal value for different ages of neoplasm probably represent inter-batch variations in stain uptake and could be reduced by expressing adenoma DNA measurements as a percentage of sperm values with both tissues on the same slide. The hyperdiploid nuclei of adenomas have about twice as much DNA as the modal value, and so probably represent tetraploid cells, in contrast to most of the adenoma nuclei which have DNA values around the diploid (2N) level, *i.e.*, about double that of the haploid sperm. The presence of normally cycling cells in S and G2 with DNA contents between 2N and 4N could complicate the interpretation of these profiles. However, the hyperdiploid nuclei appear with greater frequency as age increases whilst the proliferative activity of the adenomas decreases rapidly with age.

### DISCUSSION

The results of metaphase arrest by VCR confirm the earlier observations using Colcemid that $R_M$ is considerably less than $R_S$. The VCR $R_M$ was 0·191%/h compared with an $R_S$ of 1%/h derived by interpolation from a graph relating double labelling index to time (Dyson and Heppleston, 1975). The VCR $R_M$ is greater than the Colcemid $R_M$ of 0·113%/h formerly obtained in the same study, thus emphasizing the superiority of vincristine over Colcemid as a stathmokinetic agent. The $I_P$ value of 9% agrees with the 10% obtained by continuous labelling (Dyson and Heppleston, 1976) even though the latter method might be expected to overestimate $I_P$ if significant decycling of labelled cells was occurring. VCR was effective in arresting all cells entering mitosis, since no telophases or anaphases were found after the 75-min interval. Although it is possible that VCR prevented cells from entering mitosis, Al-Dewachi *et al.* (1975) showed that, in mouse jejunum, VCR produced kinetic results closely comparable with those obtained in labelling studies.

The present estimate of cell loss, 52%, is nearer to the 31% determined from the fraction-labelled-mitoses study, than to the figure of 83–95% given by the double labelling $R_S$ values. It therefore appears that some cell loss takes place during the growth of adenomas, although the extent is difficult to quantify. The presence of isolated pyknotic, karyorrhexic nuclei in the adenomas raised the possibility of apoptosis or spontaneous cell death (Kerr, Wyllie and Currie, 1972). Degenerate cells were noted ultrastructurally in the neoplasm (Snyder *et al.*, 1973). If cell death is cycle-specific and located somewhere in the S or G2 phases, it might explain, in part at least, the disparity between $R_S$ and $R_M$. The discovery of cells with a hyperdiploid DNA content, however, indicates that some cells entering S do not proceed to complete mitosis and yet persist. This phenomenon has previously been noted in organ cultures of normal mouse lung and prostate, where $R_S$ exceeded $R_M$ two- to four-fold, and the lung tissue exhibited a high proportion of hyperdiploid nuclei (Simnett and Heppleston, 1968).

Compared with normal tissues, a wide scatter of microspectrophotometric values has been found in human carcinomas and sarcomas, a phenomenon also apparent in some metaplastic or precancerous tissues (Leuchtenberger, Leuchtenberger and Davis, 1954; Atkin and Richards, 1956; Reid and Singh, 1960; Stich and Steele, 1962). In Sandritter's (1965) series of human malignant tumours, most showed hyperploidy, which persisted through all stages of invasion in cervical carcinoma and even to metastasis in pulmonary alveolar-cell carcinoma. Metaplastic and atypical epithelium of the human bronchus, however, had the normal pattern of DNA values, as distinct from the hyperploidy with a wide scatter of values seen in squamous carcinoma from the same cases (Sandritter *et al.*, 1965). Cervical scrapings of atypical appearance also possessed DNA values similar to those of a growing diploid population, whilst malignant and premalignant cells generally showed hyperploidy with a wide scatter (Caspersson, 1964). Hyperploidy, similar in nature to that of pulmonary adenomas but exaggerated in degree, was a feature of a rapidly growing, transplantable mouse sarcoma arising spontaneously in this laboratory. Induced transplantable ependymoma and rhabdomyosarcoma of mice showed increases in DNA that followed both a geometric series and intermediate values (Ogawa *et al.*, 1959). Disparity between $R_S$ and $R_M$ could account in part for these densitometric findings, and the changes might be more pronounced in malignant or premalignant states than under benign, atypical or normal conditions. Polyploidy alone might be expected to shift the mode only to the right, whereas spread of DNA profiles on either side of the normal diploid value for the tissue might be initiated by asymmetric mitosis.

The higher levels of ploidy seem inconsistent with the capacity to proliferate and, when predominant in human tumours, might suggest a better prognosis. Atkin (1976) has, however, shown that a raised modal level of ploidy is not necessarily a good prognostic index for human carcinomas of different origins. Squamous carcinoma of the uterine cervix carried a more favourable outlook when the mode was elevated, whereas endometrial, ovarian and breast carcinomas had a better prognosis when the mode was near-diploid. Fractionation by size or density of cells from experimental tumours or from human neoplasms that are capable of xenogeneic transplantation, with the establishment of proliferative indices in transplants from each category of ploidy, may illuminate what appears to be a further disparity (Aherne, 1976; personal communication), in which it seems that endocrine dependence may be a factor.

Circumstantial evidence of polyploidy is provided by the occurrence of many binucleate cells in the adenomas from an early stage of their development. Binuclear hepatocytes are recognized to be precursors of polyploidy, which in the rat liver apparently arises by the following sequence of events (Bucher and Malt, 1971). During postnatal development a high percentage of hepatocytes, which at birth are all mononucleate diploids, become binuclear through failure of cytoplasmic separation following nuclear division. When binucleate cells enter mitosis, the chromosomes in each nucleus double, but all deploy on a single spindle yielding two mononucleate daughter cells of the next higher order of ploidy and of correspondingly greater size. Polyploidy of the liver does not evolve in the absence of binucleate cells, nor in the absence of cell proliferation. The volume of each hepatocyte is directly proportional to its ploidy and, since the nuclear-cytoplasmic ratio is fixed, the result of increased ploidy is a relative decrease in cell surface area, the functional significance of which is unknown. In urethane-induced pulmonary adenomas the many binucleate cells, which were often more frequent than mitotic cells, could be precursors of polyploid cells. Their formation may be associated with elevation of metabolic activity, a possibility which gains credence from the large amounts of PAS-positive material found intra- and extra-cellularly in mature neoplasms. Such material may reflect the presence of lung surfactant, essentially a product of type II epithelial cells from which adenomas derive, since dipalmitoyl lecithin is synthesized by homogenates (Snyder *et al.*, 1973). Whether the metabolic turnover of this compound is augmented requires biochemical assessment by means such as those employed in elucidating the pathogenesis of experimental alveolar lipoproteinosis (Heppleston, Fletcher and Wyatt, 1974).

This work was supported by grants awarded to A.G.H. and Dr W. Aherne by the North of England Council of the Cancer Research Campaign.

### REFERENCES

AL-DEWACHI, H. S., WRIGHT, N. A., APPLETON, D. R. & WATSON, A. J. (1975) Cell Population Kinetics in the Mouse Jejunal Crypt. *Virchows Arch. B Cell Path.*, **18**, 225.

ATKIN, N. B. (1976) Prognostic Significance of Ploidy Level in Human Tumours. I. Carcinoma of the Uterus. *J. natn. Cancer Inst.*, **56**, 909.

ATKIN, N. B. & RICHARDS, B. M. (1956) Deoxyribonucleic Acid in Human Tumours as Measured by Microspectrophotometry of Feulgen Stain: a Comparison of Tumours Arising at Different Sites. *Br. J. Cancer*, **10**, 769.

BUCHER, N. L. R. & MALT, R. A. (1971) *Regeneration of Liver and Kidney*. Boston: Little Brown and Co., p. 42.

CASPERSSON, O. (1964) Quantitative Cytochemical Studies on Normal, Malignant, Premalignant and Atypical Cell Populations from the Human Uterine Cervix. *Acta cytol.*, **8**, 45.

DYSON, P. & HEPPLESTON, A. G. (1975) Cell Kinetics of Urethane-induced Murine Pulmonary Adenomata: I. The Growth Rate. *Br. J. Cancer*, **31**, 405.

DYSON, P. & HEPPLESTON, A. G. (1976) Cell Kinetics of Urethane-induced Murine Pulmonary Adenomata: II. The Growth Fraction and Cell Loss Factor. *Br. J. Cancer*, **33**, 105.

HEPPLESTON, A. G., FLETCHER, K. & WYATT, I. (1974) Changes in Composition of Lung Lipids and Turnover of Dipalmitoyl Lecithin in Experimental Alveolar Lipo-proteinosis Induced by Inhaled Quartz. *Br. J. exp. Path.*, **55**, 384.

KERR, J. F. R., WYLLIE, A. H. & CURRIE, A. R. (1972) Apoptosis: a Basic Biological Phenomenon with Wide-ranging Implications in Tissue Kinetics. *Br. J. Cancer*, **26**, 239.

LEUCHTENBERGER, C., LEUCHTENBERGER, R. & DAVIS, A. M. (1954) A Microspectrophotometric Study of the Desoxyribose Nucleic Acid (DNA) Content in Cells of Normal and Malignant Human Tissues. *Am. J. Path.*, **30**, 65.

OGAWA, K., HIMES, M., POLLISTER, A. W. & ZIMMERMAN, H. M. (1959) Changes in Deoxyribonucleic Acid Content of Experimental Tumours in C3H Mice. *Cancer Res.*, **19**, 596.

REID, B. L. & SINGH, S. (1960) Deoxyribonucleic Acid Values (Feulgen Microspectrophotometry) in Epithelium of Human Ectocervix, Normal and Cancerous. *J. natn. Cancer Inst.*, **25**, 1291.

SANDRITTER, W. (1965) DNA Content of Tumours: Cytophotometric Measurements. *Eur. J. Cancer*, **1**, 303.

SANDRITTER, W., SEIDEL, A., KLEINHANS, D., PADDAGS, I. & DONTENWILL, W. (1965) Cytophotometrische Messungen des DNA-Gehaltes an menschlichen und tierexperimentellen Bronchialepithelmetaplasien. *Z. Krebsforsch.*, **67**, 69.

SIMNETT, J. D. & HEPPLESTON, A. G. (1968) Rates of Deoxyribonucleic Acid Synthesis in Organ Cultures of Lung and Prostate as Measured by Tritiated Thymidine Autoradiography. *Lab. Invest.*, **19**, 333.

SMITH, S. R., THOMAS, D. B. & RICHES, A. C. (1972) Metaphase Accumulation in Tumour Isografts Following Administration of Colcemid, Vinblastine or Vincristine. *J. Anat.*, **111**, 480.

SMITH, S. R., THOMAS, D. B. & RICHES, A. C. (1974) Cell Production in Tumour Isografts Measured Using Vincristine and Colcemid. *Cell Tissue Kinet.*, **7**, 529.

SNYDER, C., MALONE, B., NETTESHEIM, P. & SNYDER, F. (1973) Urethan-induced Pulmonary Adenoma as a Tool for the Study of Surfactant Biosynthesis. *Cancer Res.*, **33**, 2437.

STEEL, G. G. (1968) Cell Loss from Experimental Tumours. *Cell Tissue Kinet.*, **1**, 193.

STICH, H. F. & STEELE, H. D. (1962) DNA Content of Tumour Cells. III. Mosaic Composition of Sarcomas and Carcinomas in Man. *J. natn. Cancer Inst.*, **28**, 1207.

TANNOCK, I. F. (1967) A Comparison of the Relative Effectiveness of Various Metaphase Arrest Agents. *Expl Cell Res.*, **47**, 345.